# Considerations for a Micromirror Array Optimized for Compressive Sensing (VIS to MIR) in Space Applications

**DOI:** 10.3390/jimaging10110282

**Published:** 2024-11-05

**Authors:** Ulrike Dauderstädt, Peter Dürr, Detlef Kunze, Sara Francés González, Donato Borrelli, Lorenzo Palombi, Valentina Raimondi, Michael Wagner

**Affiliations:** 1 Fraunhofer IPMS, Maria-Reiche-Straße 2, 01109 Dresden, Germany; peter.duerr@ipms.fraunhofer.de (P.D.); sara.frances@ipms.fraunhofer.de (S.F.G.); michael.wagner@ipms.fraunhofer.de (M.W.); 2 LEONARDO S.p.A., 50013 Campi Bisenzio, Italy; donato.borrelli@leonardocompany.com; 3 IFAC-CNR, 50019 Sesto Fiorentino, Italy; l.palombi@ifac.cnr.it (L.P.); v.raimondi@ifac.cnr.it (V.R.)

**Keywords:** micromirror array, spatial light modulator, compressive sensing

## Abstract

Earth observation (EO) is crucial for addressing environmental and societal challenges, but it struggles with revisit times and spatial resolution. The EU-funded SURPRISE project aims to improve EO capabilities by studying space instrumentation using compressive sensing (CS) implemented through spatial light modulators (SLMs) based on micromirror arrays (MMAs) to improve the ground sampling distance. In the SURPRISE project, we studied the development of an MMA that meets the requirements of a CS-based geostationary instrument working in the visible (VIS) and mid-infrared (MIR) spectral ranges. This paper describes the optical simulation procedure and the results obtained for analyzing the performance of such an MMA with the goal of identifying a mirror design that would allow the device to meet the optical requirements of this specific application.

## 1. Introduction

Earth observation (EO) data are becoming increasingly important to our understanding of our planet and to addressing societal and environmental challenges. Applications include the mapping and characterization of Earth surfaces for environmental monitoring (from forest biodiversity to refugee camps), the monitoring of natural and human-induced risks (e.g., floods, fires, and pollution), and services in agriculture and aquaculture. Two important challenges to the implementation of EO from space are revisit times and spatial resolution. While low Earth observation (LEO) missions can achieve resolutions better than 100 m, their revisit time typically stands at several days, limiting their capacity to monitor dynamic events and risks. Geostationary (GEO) missions, on the other hand, provide data on an hourly basis, but their spatial resolution is currently limited to 1 km typically, which is insufficient for understanding local phenomena.

This challenge is addressed with a new technical approach carried out in the SURPRISE project, funded by the European Union, which aims at a concept for a space instrument with a medium ground sampling distance (a few hundred meters) for the visible (VIS) and near (NIR) and mid-infrared (MIR) spectral ranges [1]. This goal will be achieved using compressive sensing (CS) technology implemented via spatial light modulators (SLMs) based on micromirror arrays (MMAs). SLM-based CS technology will be used to devise a super-resolution configuration that will be exploited to increase the at-ground spatial sampling of the payload without increasing the number of the detector’s sensing elements at the image plane.

In this paper, we will describe an MMA that is suited to this application and present an analysis of the performance expected, given the specifications of the optical system. The novelty of this work is based especially on the analysis for the MIR.

## 2. Micromirror Arrays for Compressive Sensing

Compressive sensing is a method that allows the acquisition of high-resolution images by means of a single-pixel detector or a low-resolution camera in combination with a high-resolution SLM. The SLM is used to implement several modulation patterns (coding masks), which are applied sequentially to the image focused on the SLM plane. The modulated image is then integrated via a condenser, and the resulting integrated signal is recorded via the single-pixel detector. Finally, the original image is reconstructed using suitable CS reconstruction algorithms; see [2,3,4].

**Figure 1:**
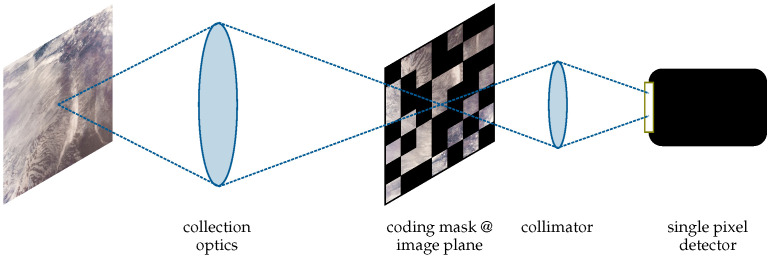
Compressive sensing principle.

The basic principle of CS using an SLM is illustrated in Figure 1, [5,6]:

- A collection optics collects the light coming from the target
- An SLM physically implements the scalar product between the coding mask applied to the SLM and the image focused, via the collection optics, on the SLM plane.
- An optical condenser implements the optical integration.
- A single-element detector or spectral demultiplexer is used to acquire the signal.

**Figure 2:**
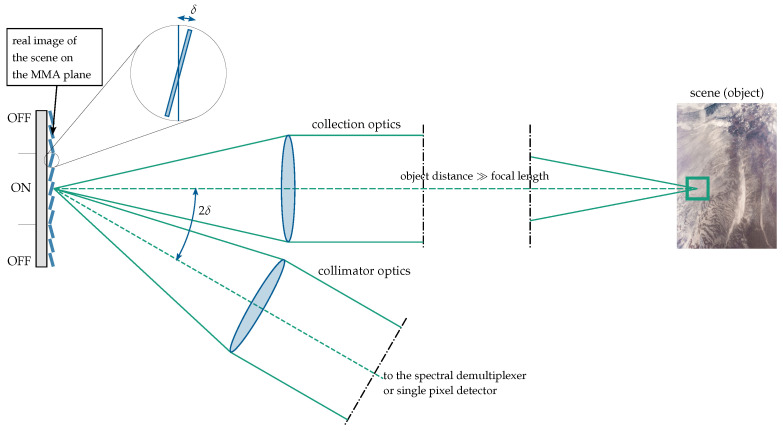
Schematic illustration of the optical principle.

If the SLM is a micromirror array, it is operated in a reflective mode, as shown in Figure 2. The collection optics create a real image of the scene in the MMA plane, where the coding mask is applied. The light from the mirrors in the ‘ON’ state is reflected into the collimator optics (which also acts as an optical condenser) and the detector. In order to take into account diffraction effects that can become particularly important when dealing with IR light, a wave optics approach is required.

A major parameter to analyze is the efficiency of the MMA at a given wavelength, i.e., the portion of light that reaches the detector from a pixel in the ‘ON’ state, as well as that from a pixel in the ‘OFF’ state, where a ‘pixel’ can be a single micromirror or several mirrors combined into one logical pixel; see Section 5.6. The ratio between the ‘ON’ and ‘OFF’ efficiencies should be as high as possible. The influence of the parameters defining the mirrors on the efficiencies will be explored in Section 5.4.3.

In the following, we will discuss several aspects to define the micromirrors’ specifications:

The actuation mode of the mirrors: The mirror architecture will be described, and two alternative actuation modes will be discussed in relation to its application for compressive sensing in space.

Illumination: The SLM, as an optical grating, the blaze condition, and the SLM illumination will be discussed.

Tilt direction: Two versions of tilting mirrors will be discussed, diagonal mirrors tilted around the diagonal and orthogonal mirrors, which are tilted around an axis parallel to an edge.

Optical model: The algorithms used for the simulation are described, starting with the illumination, the description of the mirrors, and the Fourier transform; finally, the description of the efficiency will be discussed.

The influence of mirror and system parameters on performance: the influence of mirror size, mirror deflection, and the f-number of the optics will be discussed, as well as the effect of binning, i.e., the combination of serveral mirrors into one logical pixel.

## 3. Digital Micromirror Devices by Texas Instruments

In the last decades, many micromirror devices have been developed for a multitude of applications (see [7] for a comprehensive overview). However, there are only relatively few devices commercially available that are also suitable for operation in the infrared spectral range. The most prominent of these are the digital mirror devices (DMD) by Texas Instruments. These DMDs have been used in most works on compressive sensing so far. In principle, these devices are very well suited to this application due to their small size and high speed; see [2,3,4].

However, only very few of these devices can operate in the infrared range, and usually, they are not suitable for operation beyond the short-wavelength infrared (SWIR) spectral range. In addition to their window, which is not transparent to IR light and needs to be replaced (see [8]), their deflection is insufficient for larger wavelengths. (The relation between the mirror size, deflection angle, and deflection is explained in more detail in Section 5.3). For example, the DLP©7000, see [9], which comes very close to meeting the requirements of this application, has a mirror size of 13.68 μm and a deflection angle of 12°. This leads to a blaze wavelength of about 4 μm, which would barely meet our current requirements. One aim of this work is to investigate what would be required to cover the full MIR spectral range. In addition, in order to avoid any risk of micromirror sticking and increase reliability, we consider using an analog deflection mode instead of a mechanical stop actuator, as used in DMD devices; see Section 4.

## 4. Mirror Actuation Mode: Contact vs. Non-Contact

**Figure 3:**
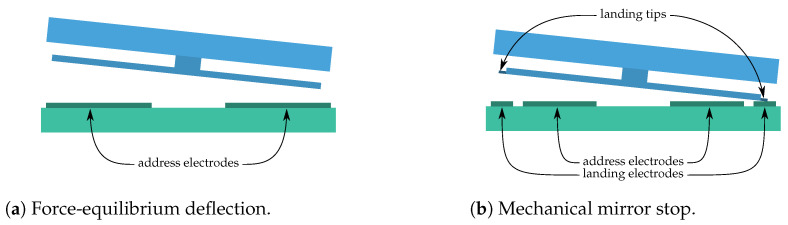
Mirror actuation modes.

Electrostatic actuation is one of the most popular mechanisms used in microelectromechanical systems (MEMSs) because of its flexible operation and easy fabrication from standard and well-understood materials [10]. During the SURPRISE project, the Fraunhofer Institute for Photonic Microsystems (IPMS) evaluated two different actuator types, each with several advantages and disadvantages for space applications: force equilibrium deflection or a non-contact deflection mode and a mechanical mirror stop, with which the mirror makes contact with a landing electrode. Both are depicted in Figure 3 and described below.

**Figure 4:**
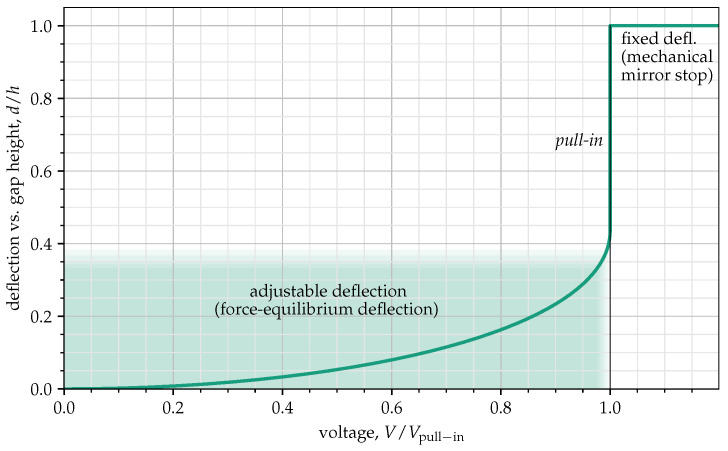
Mirror deflection vs. addressing voltage.

While, for the mechanical stop option, two surfaces contact each other, defining a fixed tilt angle, the force equilibrium option is a contact-less actuator for which the tilt angle can be adjusted in a certain range by modifying the addressing voltage. Figure 4 shows this aspect: the adjustable range of the tilt curve corresponds to the possible range for a force equilibrium deflection actuator, and the fixed angle range shows the range at which the mechanical stop actuator works. The tilt angle achieved in this case is always fixed and depends only on the geometric layout, i.e., the relation between the mirror size and the actuator gap height. This choice leads to notable consequences: with a mechanical mirror stopping mode, it is possible to achieve a fixed maximum tilt angle and deflect the mirror in two opposite directions; in what is called binary mode, only two deflection states are possible. On the other side, the force equilibrium deflection mode allows addressing micromirrors in a continuous deflection (analog) mode. This means a micromirror can be deflected not only to two binary states but also to many different intermediate tilt angles. However, this actuation scheme results in highly non-linear dynamics, giving rise to pull-in phenomena, which limits the stable open-loop operation to a small portion of the whole physically available range [11].

Both modes can be addressed with very similar actuators except that, in the non-contact case, the gap height needs to be larger in order to reach the same deflection angle. Also, each mode requires a corresponding MMA driver system. Typically, an MMA with its driver is designed for only one actuation mode.

Mechanical mirror stopping in a MEMS actuator, as it is provided by Texas Instruments Devices [12], offers some advantages in space applications: the deflection angle is well defined, and future calibrations of the mirror deflection are definitely not needed. Due to the fixed angle, it may be more robust, for example, if the damping changes.

Force equilibrium deflection mode, on the other hand, will avoid contacting surfaces and, therefore, non-desired sticking effects. Sticking occurs because microscopic structures tend to adhere to each other when they come into contact. This is because the structures are so small that surface forces can dominate all other effects. Avoiding sticking is, therefore, beneficial in terms of reliability, allowing a longer SLM lifetime and matching quite well with space application requirements [13].

In addition, many good results in performance were demonstrated by IPMS MMA using force equilibrium deflection mode (analog mode) for different applications; for an overview, see [14], in some of which the force equilibrium was used mostly for grayscaling and for wavelength adaptation [15].

A summary of both deflection modes is outlined in Table 1.

## 5. Theoretical Investigations for the New Micromirror Array

Here, we will discuss the specifications of the SLM as they were posed in a pre-feasibility study of a CS-based optical payload operating in the VNIR and MIR from geostationary platform created by the SURPRISE Consortium. We will pay particular attention to the limitations posed in the micromirror design and the resulting optical performance.

### 5.1. System Specifications and Their Impact on the Mirror Design

The system specifications on which the calculations in this paper are based are presented in Table 2.

Since the SLM is positioned in the focal plane of the collection optics, and each data point on the scene that is to be observed should correspond to at least one pixel of the SLM, the ground sampling distance (GSD) is related to the pixel size of the SLM (*p*) as follows:

(1) $$\frac{GSD}{h_{\mathrm{orbit}}}=\frac{p}{f_{\mathrm{collect}}}$$

with the orbit height ($h_{\mathrm{orbit}}$) and the collection optics focal length ($f_{\mathrm{collect}}$). For a low value of GSD, low values for *p* and high values for $f_{\mathrm{collect}}$ would be preferable. On the other hand, the maximum size of $f_{\mathrm{collect}}$ is limited due to the requirements for the size and mass of the payload.

From this, it follows that the maximum pixel pitch is

(2) $$p_{\max}=\frac{GSD_{\max}f_{\mathrm{collect},\max}}{h_{\mathrm{orbit}}}$$

With a given collection optics diameter ($d_{\mathrm{collect}}$), we can calculate the minimum pixel pitch

(3) $$p_{\min}=\frac{GSD_{\max}N_{\mathrm{collect},\min}d_{\mathrm{collect}}}{h_{\mathrm{orbit}}}$$

where

(4) $$N_{\mathrm{collect}}=f_{\mathrm{collect}}/d_{\mathrm{collect}}$$

is the f-number of the collection optics. From an optical efficiency point-of-view, the f-number should be small to allow fast optics; see Section 5.4.3. If it is too small, however, the image is affected by aberrations.

Further restrictions on the minimum pitch are posed by the deflection angle, which is required to satisfy the blaze condition at a given wavelength ($\lambda_{B}$). If the deflection angle is too high, the voltage required for deflection will be too high as well. Since a smaller pitch requires a larger deflection angle, it might be necessary to increase the pitch in order to achieve the necessary deflection at higher wavelengths.

In addition, the distance between the mirror and an electrode will have to be larger in order to achieve larger angles, making the fabrication of the device more complex and increasing the required voltage even further. Finally, for large angles, the condition cosδ≈1 is no longer satisfied, leading to a smaller effective electrode area (i.e., smaller electrostatic forces) and larger slits: a minimal but still necessary gap between the sides of two contiguous mirrors. (Note: in the calculations below, the influence of the slits is ignored, which is legitimate if the slit width is small compared to the wavelength).

In the SURPRISE project, we address the range from the visible-near infrared (VNIR) up to the mid-infrared (MIR) spectral range (up to wavelengths of at least 4 μm), with the higher wavelengths being the most challenging ones, especially in terms of diffraction.

Note that, in this section, pixel pitch does not necessarily mean mirror pitch, but (theoretically) several mirrors can be combined (binned) to form a logical pixel, thus achieving larger pixels with small mirrors; see also Section 5.6. However, in most calculations in this paper, no binning was used, and therefore, pixel pitch and mirror pitch are the same unless stated differently.

Table 2 provides an overview of the basic system specifications for the payload in the SURPRISE project and the resulting range for the pixel size. Here, the maximum value for the GSD is given as 500 m, which was the initial limit discussed. However, during the project, it turned out that a smaller value would be desirable. This is discussed in Section 5.7.

### 5.2. Illumination

**Figure 5:**
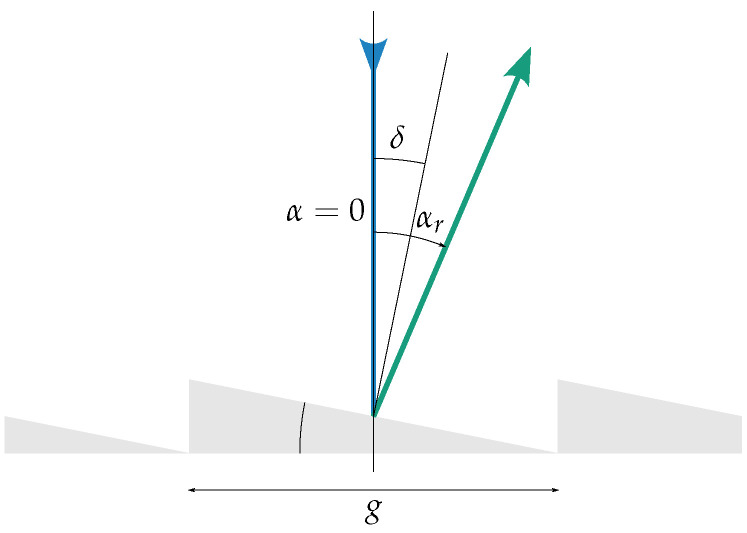
On-axis illumination–angles.

The concept considered for the SURPRISE project is based on an on-axis illumination (see Figure 5), where the incident light is at a right angle to the SLM plane; i.e., the angle between the incident light and the normal of the SLM plane, α, is zero.

The SLM acts like optical grating. Its blaze wavelength [18] ([pp. 947–962]), [19], which depends on the grid constant (*g*) and deflection angle (δ), forms a theoretical upper limit to the operational spectral range.

The blaze wavelength for on-axis illumination is given by

(5) $$\lambda_{B}=g\sin2\delta$$

The grid constant depends on the mirror pitch and deflection mode, and it is discussed in the next section.

### 5.3. Tilt Deflection Mode: Diagonal vs. Orthogonal

**Figure 6:**
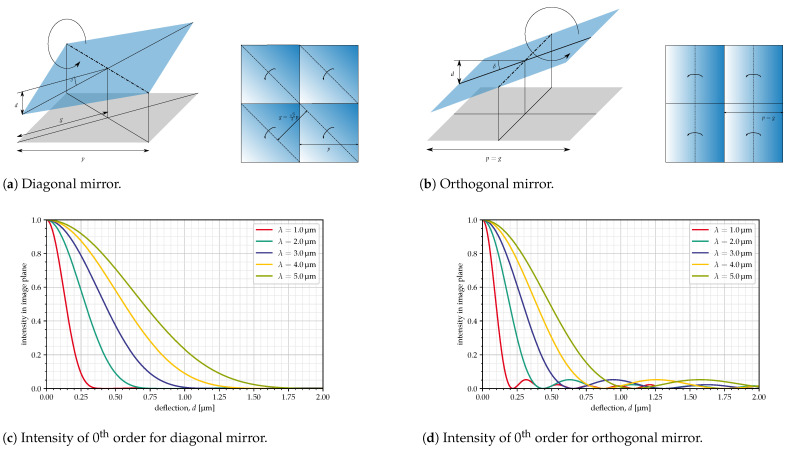
Diagonal vs. orthogonal mirror.

Two different deflection modes were evaluated from the point of view of contrast and deflection required for the infrared spectral range (λ up to at least 4 μm): diagonal mode (working mode of the Texas Instruments DLP©7000, [9]) and orthogonal mode, where the tilt axis is parallel to the mirror edge; see Figure 6. The latter is not to be confused with the tip-and-roll mode (TRP) of the DLP©2010NIR by Texas Instruments [20], where the ‘ON’ and ‘OFF’ states of the mirror are not to the left and right of the axis of rotation but to the left and the bottom of the pixel.

For square mirrors, which are tilted around an axis parallel to an edge, the grid constant, *g*, is identical to the mirror pitch; for diagonal mirrors, it is reduced to $p/\sqrt{2}$ (the distance between the mirrors’ axes of rotation, i.e., half the mirror diagonal), as indicated in Figure 6a,b.

The deflection angle (δ) required to satisfy the blaze condition at $\lambda_{B}$ from Equation (5) becomes, for diagonal mirrors,

(6) $$\delta =\frac{1}{2}\arcsin\frac{\sqrt{2}\lambda_{B}}{p}$$

and for orthogonal mirrors

(7) $$\delta =\frac{1}{2}\arcsin\frac{\lambda_{B}}{p}$$

The resulting mirror deflection (*d*) for diagonal mirrors, where the deflection is the height difference between the mirror center and corner, is

(8) $$d=\frac{\sqrt{2}}{2}p\sin\delta$$

And for orthogonal mirrors, where the deflection is the height difference between mirror center and edge, it is

(9) $$d=\frac{p}{2}\sin\delta$$

Table 3 shows the deflections for the mirror sizes from Table 2 at a blaze wavelength of 4 μm. However, there are some limitations on mirror size and deflection resulting from the electrostatic addressing of the mirrors. The mirror deflection that can be achieved depends on the mirror pitch (a large pitch allows for larger electrodes and, therefore, larger electrostatic forces), the gap height between mirror and the electrode (which needs to be large enough to allow the desired deflection, but a larger gap heights result in smaller electrostatic forces), and the available voltage, which is limited by the CMOS process used for fabrication. In addition, a real mirror array will have slits between the mirrors (with a width of a few hundred nanometers), which are neglected in the calculations in this paper, but it will have a proportionally larger influence on the performance if the mirrors are smaller. It can be seen from the table that, for the smallest pixel sizes, the angles become rather large, which, for these reasons, would be difficult to achieve, so they may not be usable, especially if one wants to further extend the wavelength range to 5 μm or more.

The intensity curves in Figure 6c,d, obtained via a Fourier transform of the phase profile in the mirror plane, are those for the zeroth diffraction order if all mirrors are set to the same deflection and the influence of the collection optics is neglected.

For orthogonal mirrors, this intensity is given by

(10) $$I={\mathrm{sinc}}^{2}\frac{4\pi d}{\lambda }$$

while it is

(11) $$I={\mathrm{sinc}}^{4}\frac{2\pi d}{\lambda }$$

for diagonal mirrors, where the areas with the largest displacement, near the corner of the mirror, contributes much less to the total intensity than the areas with the smallest displacement, near the axis of rotation. This explains the larger deflection required in comparison to the orthogonal mirrors, for which all areas contribute equally. For more details on the derivation of these equations, see [21,22].

It can also be seen that, for diagonal mirrors, once the nominal deflection is reached (where the blaze condition is satisfied for the given wavelength), the intensity remains near zero; meanwhile, for orthogonal mirrors, there are several local maxima for the intensity at larger deflections. The reason for this is that, with the fourth power of the sinc-function for the diagonal mirrors, the effect of larger deflections is much reduced in comparison to the orthogonal mirrors.

This is an indication that diagonal mirrors may be better suited to the current application. For very small wavelengths this is not so important, but for wavelengths in the range of about $0.5\lambda_{B}<\lambda <\lambda_{B}$, pixels in the ‘OFF’ state would not block all the light but would let through up to 5% in the intensity of pixels in the ‘ON’ state. On the other hand, orthogonal mirrors require smaller deflections to reach the same blaze wavelength, which is an advantage with respect to the fabrication of the mirrors because it would mean that a smaller voltage and a smaller gap height would be required.

From these considerations, and from the fact that it would be preferable if the mirrors were usable not only for this specific application but also for other applications, it would follow a preference for diagonal mirrors with a pitch close to the upper limit. Therefore, we will mainly investigate mirror sizes in the range of about 16 μm to 30 μm, which would be safely inside the possible range for the pixel pitch and would require reasonable deflection angles.

### 5.4. Optical Model

In the following, we explain the model for the optical simulations. The calculations, as well as the creation of the figures, were carried out in Python (version 3.11.5).

#### 5.4.1. Illumination

The impact of each point in the scene to be observed on the mirror array can be described using an Airy disk whose shape is defined by the wavelength (λ) and the f-number of the collection optics ($N_{\mathrm{collect}}$). It is described by a Bessel function ([18] (pp. 931–939), [23]):

(12) $$E_{\mathrm{Airy}}=E_{0}\frac{2J_{1}\left(x\right)}{x}$$

with

(13) $$x=\frac{kaq}{R}\approx \frac{\pi }{\lambda }\frac{q}{N_{\mathrm{collect}}}$$

where

*k* — wave number, k=2π/λ;
*q* — radial distance at SLM plane;
*R* — distance from collection lens to position on SLM, $R\approx f_{\mathrm{collect}}$;
*a* — half the collection optics diameter, $a=d_{\mathrm{collect}}/2$.

The amplitude at the center of the diffraction pattern ($E_{0}$) is related to the total power incident on the aperture ($P_{0}$):

(14) $$E_{0}=\frac{P_{0}A}{\lambda^{2}R^{2}}$$

where *A* is the area of the aperture ($A=\pi d_{\mathrm{collect}}^{2}/4$), and *R* is the distance from the aperture.

At the focal plane of a lens,

(15) $$E_{0}=\frac{P_{0}\pi d_{\mathrm{collect}}^{2}}{4\lambda^{2}f_{\mathrm{collect}}^{2}}$$

The diameter of the Airy disk on the SLM surface (defined by the first zero of Equation (12)) is, then,

(16) $$D_{\mathrm{Airy}}\approx 2.44\lambda N_{\mathrm{collect}}$$

**Figure 7:**
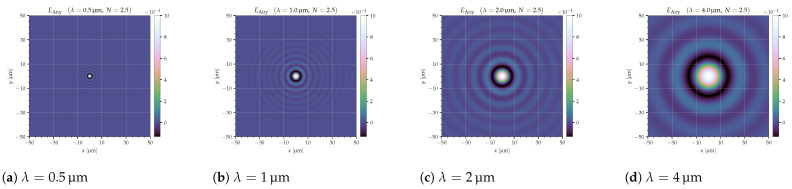
Airy disks for different wavelengths (amplitude, $E_{\mathrm{Airy}}$). The mirror size in this example is 20 μm, as indicated by the gridlines.

Figure 7 shows four examples of Airy disks for the wavelengths 0.5 μm, 1 μm, 2 μm, and 4 μm. The grid lines indicate mirrors of pitch 20 μm. It can be seen that, for large wavelengths, the Airy disk becomes larger than one mirror, which would result in a lower efficiency.

In the following simulations, the efficiency was calculated for one logical pixel (single mirror or several binned mirrors) in the ‘ON’ or ‘OFF’ state surrounded by mirrors in the opposite state. In this way, it can be determined how much of the desired Airy disk is detected (‘ON’) or blocked (‘OFF’).

#### 5.4.2. Mirror Profile and Fourier Transform

**Figure 8:**
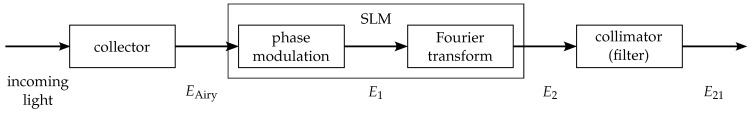
Optical signal flow.

Figure 8 shows the optical signal flow in the system. The Airy disk created via the collection optics is phase-modulated and Fourier-transformed at the SLM. Finally, the intensity in the Fourier space is filtered by the collimator. In the following, this is described in detail.

The height profile in the mirror plane is described by

(17) $$z\left(x,y\right)=-\frac{d}{p}\;\mathrm{mod}\;x-\frac{p}{2}\;\mathrm{mod}\;n_{x},2,p-\frac{d}{p}\;\mathrm{mod}\;y-\frac{p}{2}\;\mathrm{mod}\;n_{y},2,p-d$$

for diagonal mirrors and

(18) $$z\left(x,y\right)=-\frac{2d}{p}\;\mathrm{mod}\;x-\frac{p}{2}\;\mathrm{mod}\;n_{x},2,p-d$$

for orthogonal mirrors, where $n_{x}$ and $n_{y}$ give the number of mirrors (in the *x*- and *y*-directions) combined into a logical pixel. For most examples here, only one mirror per pixel is used ($n_{x}=n_{y}=1$), except in Section 5.6, where the effect of using binned mirrors is investigated.

From this, the phase of the reflected light is derived:

(19) $$\varphi \left(x,y\right)=-\frac{4\pi z}{\lambda }+\varphi_{\mathrm{Airy}}\left(x,y\right)$$

The complex amplitude in the mirror plane is, then,

(20) $$E_{1}\left(x,y\right)=r\left(x,y\right)\left|E_{\mathrm{Airy}}\left(x,y\right)\right|\;\cdot \;e^{i\varphi (x,y)}$$

and the intensity

(21) $$I_{1}\left(x,y\right)={\left|E_{1}\left(x,y\right)\right|}^{2}$$

with r(x,y) being the reflectivity of the modulator. For ideal mirrors without slits, the reflectivity is the same everywhere, so it is set to one in all calculations in this paper.

The complex amplitude in the Fourier plane is obtained via a Fourier transform of $E_{1}$, [18] (pp. 1031–1118), [24]

(22) $$E_{2}\left(u_{x},u_{y}\right)=F\left\{E_{1}\left(x,y\right)\right\}$$

with the intensity

(23) $$I_{2}\left(u_{x},u_{y}\right)={\left|E_{2}\left(u_{x},u_{y}\right)\right|}^{2}$$

**Figure 9:**
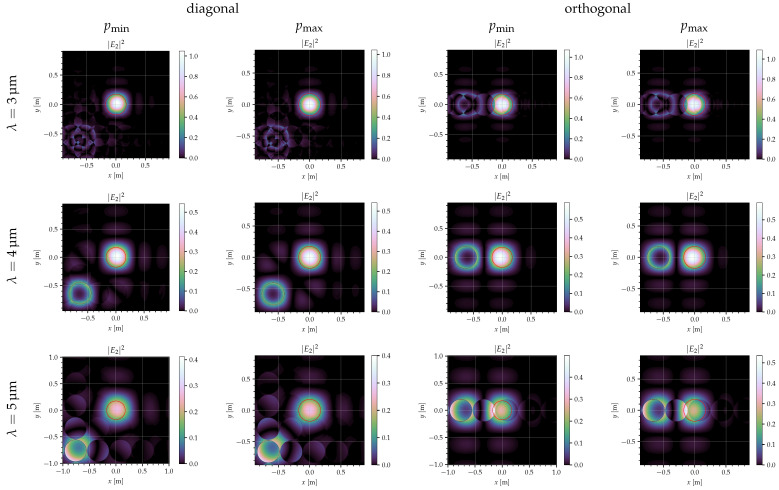
Intensity in the Fourier plane, $I_{2}\left(x,y\right)={\left|E_{2}\right|}^{2}$, for one pixel in the ‘ON’ state surrounded by pixels in the ‘OFF’ state with the configurations from Table 3. The red circles indicate the area seen via the collimator.

The coordinates in the Fourier plane are as follows:

(24) $$\begin{array}{cc} x_{2} & =f_{\mathrm{collim}}\tan\theta_{1}\left(u_{x}-u_{x0}\right) \end{array}$$

(25) $$\begin{array}{cc} y_{2} & =f_{\mathrm{collim}}\tan\theta_{1}\left(u_{y}-u_{y0}\right) \end{array}$$

with $\left[u_{x0},u_{y0}\right]$ being the coordinates of the center of the collimator lens in units of diffraction orders at $\lambda_{B}$, usually [1,1] for diagonal tilting mirrors and [1,0] for orthogonal mirrors. The result of this calculation can be seen in Figure 9 for different mirror types (diagonal/orthogonal), mirror sizes, and wavelengths.

The deflection of the mirrors was chosen such that the blaze wavelength is $\lambda_{B}=4\;\mathrm{nm}$, and the optics dimensions were adjusted according to the equations in Section 5.1 in order to meet the system specifications (see also Table 4). In all images, the light from the mirror in the ‘ON’ state is in the center, which is where the collimator is placed (marked with the red circle). In order to achieve a good signal, as much light from the mirrors in the ‘ON’ state as possible should be inside the red circle, while light from the mirrors in the ‘OFF’ state should be outside.

For the blaze wavelength, the light from the mirrors in the ‘ON’ state forms a nice circle, and the light from the mirrors in the ‘OFF’ state forms a ring. If $\lambda \neq \lambda_{B}$, the images are more complex, and the ‘ON’ and ‘OFF’ states are harder to separate. Especially for $\lambda >\lambda_{B}$, the light from the two states overlaps more and more.

For the orthogonal mirrors, the light from the ‘OFF’ mirrors is to the left of that from the ‘ON’ mirrors, for the diagonal mirrors it is to the lower left, which means the distance between the two states is larger by a factor of $\sqrt{2}$. This corresponds to the smaller grid constant of the diagonal mirrors.

Since the optics parameters were adjusted to the mirror size, the differences between the large and small mirrors are very slight. However, there are more asymmetries and irregularities for the smaller mirrors (they can be seen best in the center row), indicating that using larger mirrors might be advantageous in some situations.

Finally, the intensity of the output signal is the part of $I_{2}$ that falls inside the collimator lens:

(26) $$I_{21}=\left\{\begin{array}{cc} I_{2} & \mathrm{inside}\;\mathrm{collimator}\\ 0 & \mathrm{outside}\;\mathrm{collimator} \end{array}\right.$$

For these calculations, the collimator diameter and focal length were always chosen so that

(27) $$N_{\mathrm{collim}}=N_{\mathrm{collect}}$$

This is not a requirement, but as can be seen in Figure 9, a pixel in the ‘ON’ state lies in a roughly circular area approximately the size of the area seen via the collimator if this condition is met. Therefore, the f-numbers of the collection optics and the collimator should be similar because, if the collimator is too large, more light that is supposed to be blocked will get through; meanwhile, if the collimator is too small, light will be lost.

#### 5.4.3. Diffraction Efficiency

We define the diffraction efficiency as the portion of light that gets through the collimator divided by the total intensity of the Airy disk:

(28) $$\varepsilon =\frac{\sum I_{21}}{\sum I_{\mathrm{Airy}}}$$

The efficiency for a pixel in the ‘ON’ state, where we want as much light as possible from the Airy disk to end up inside the collimator, depends on the size of the Airy disk relative to the pixel pitch, the wavelength, and the collimator size. However, it is also important to look at the efficiency of ‘OFF’ pixels, where we want to block as much light as possible.

In the following, the efficiencies of pixels in the ‘ON’ and ‘OFF’ states are analyzed in a wavelength range from 0.4 μm to 5 μm. The input parameters are summarized in Table 4 and Table 5. These tables also include the ratio between ‘ON’ and ‘OFF’ efficiency ($\varepsilon_{w}/\varepsilon_{b}$) at λ=4 μm in order to allow a quantitative evaluation and comparison of the different versions.

**Table 4:** Parameters for all curves in Figure 10, Figure 11, Figure 12, Figure 13, Figure 14, Figure 15 and Figure 16 (violated specifications in red) (numbers are rounded to about three significant digits; whole numbers indicate that this number was input in the calculation directly, except for GSD, which is always given in whole numbers).

Parameter	Unit	Figure 10 ($p_{\min}$, $p_{\max}$)				Figure 12 (δ)				Figure 13 (p)			
type		diag	diag	ortho	ortho	diag	diag	diag	diag	diag	diag	diag	diag
*p*	[μm]	9.72	34.72	9.72	34.72	30	30	30	30	16	20	24	30
$\lambda_{B}$	[μm]	4	4	4	4	3.68	4.41	5.13	5.85	4	4	4	4
δ	[°]	17.79	4.69	12.15	3.31	5	6	7	8	10.35	8.21	6.82	5.43
*d*	[μm]	2.1	2.01	1.02	1	1.85	2.22	2.59	2.95	2.03	2.02	2.01	2.01
$D_{\mathrm{Airy}}$	[μm]	19.5	69.7	19.5	69.7	55.5	66.4	77.3	88	32.1	40.2	48.2	60.2
$D_{\mathrm{Airy}}/p$		2.01	2.01	2.01	2.01	1.85	2.21	2.58	2.93	2.01	2.01	2.01	2.01
$N_{\mathrm{collect}}$		2	7.14	2	7.14	6.17	6.17	6.17	6.17	3.29	4.11	4.94	6.17
$f_{\mathrm{collect}}$	[m]	0.7	2.5	0.7	2.5	2.16	2.16	2.16	2.16	1.15	1.44	1.73	2.16
$d_{\mathrm{collect}}$	[m]	0.35	0.35	0.35	0.35	0.35	0.35	0.35	0.35	0.35	0.35	0.35	0.35
GSD	[m]	500	500	500	500	500	500	500	500	500	500	500	500
$\varepsilon_{w}/\varepsilon_{b}\left(\lambda =4\;\mu m\right)$		4.46	3.41	5.11	3.92	3.65	3.83	4.87	5.14	3.71	3.60	3.52	3.45
**Parameter**	**Unit**	**Figure 14 (p, const. δ)**				**Figure 15 (N)**				**Figure 16 (p, const. δ, N)**			
type		diag	diag	diag	diag	diag	diag	diag	diag	diag	diag	diag	diag
*p*	[μm]	16	20	24	30	30	30	30	30	16	20	24	30
$\lambda_{B}$	[μm]	4	5	6	7.5	4.41	4.41	4.41	4.41	2.35	2.94	3.53	4.41
δ	[°]	10.35	10.35	10.35	10.35	6	6	6	6	6	6	6	6
*d*	[μm]	2.03	2.54	3.05	3.81	2.22	2.22	2.22	2.22	1.18	1.48	1.77	2.22
$D_{\mathrm{Airy}}$	[μm]	60.2	75.3	90.3	112.9	30.7	46.1	61.5	76.9	35.4	44.3	53.1	66.4
$D_{\mathrm{Airy}}/p$		3.76	3.76	3.76	3.76	1.02	1.54	2.05	2.56	2.21	2.21	2.21	2.21
$N_{\mathrm{collect}}$		6.17	6.17	6.17	6.17	2.86	4.29	5.71	7.14	6.17	6.17	6.17	6.17
$f_{\mathrm{collect}}$	[m]	2.16	2.16	2.16	2.16	1	1.5	2	2.5	2.16	2.16	2.16	2.16
$d_{\mathrm{collect}}$	[m]	0.35	0.35	0.35	0.35	0.35	0.35	0.35	0.35	0.35	0.35	0.35	0.35
GSD	[m]	267	333	400	500	1080	720	540	432	267	333	400	500
$\varepsilon_{w}/\varepsilon_{b}\left(\lambda =4\;\mu m\right)$		3.71	4.75	5.12	3.78	11.28	9.78	5.24	2.03	0.15	1.05	2.27	3.78

**Figure 10:**
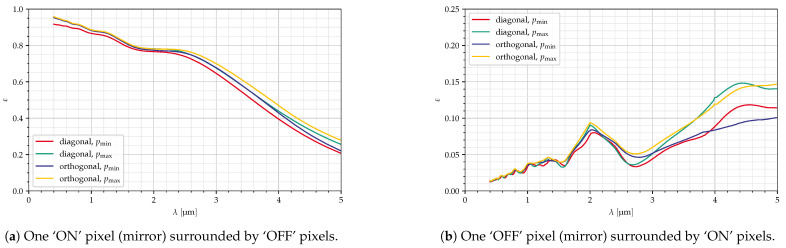
Efficiency for ‘ON’ and ‘OFF’ pixels, minimum and maximum mirror size (Table 3), all parameters in Table 4.

Figure 9 and Figure 10 show the intensity in the Fourier plane and efficiencies for the configurations from Table 3. The red circles in Figure 9 indicate the area seen via the collimator. The deflection angles are chosen so that the blaze wavelength is 4 μm in all cases. Therefore, the curves are quite similar, although the efficiencies are slightly better for the orthogonal mirrors. Also, at large wavelengths, the efficiencies (both for ‘ON’ and ‘OFF’ pixels) are slightly larger for large mirrors, resulting, however, in a smaller value for $\varepsilon_{w}/\varepsilon_{b}$.

**Figure 11:**
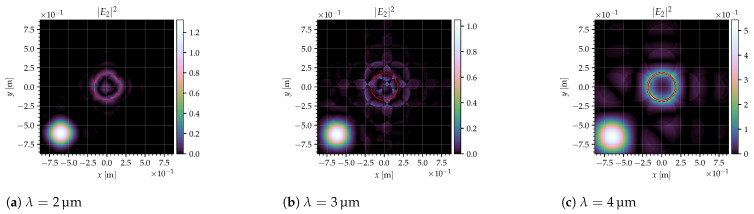
Intensity, $I_{2}={\left|E_{2}\right|}^{2}$ in collimator plane for ‘OFF’ pixel with $p=p_{\min}$, diagonal mirror.

For the ‘OFF’ pixel, an interesting peak can be seen at 2 μm. This is caused by the specific pattern of the light at different wavelengths. As can be seen in Figure 11, at 2 μm, which is half the blaze wavelength, the light in and around the collimator (indicated with the red circle), which is the light that is outside the pixel under consideration, almost forms a ring, which lies on the outer edge of the collimator; meanwhile, at 3 μm, this light forms a more complex pattern, and more light lies outside the collimator. If the collimator f-number would be slightly increased (by reducing $d_{\mathrm{collim}}$ or increasing $f_{\mathrm{collim}}$), this peak may be reduced significantly with only a small loss in the ‘ON’ efficiency.

### 5.5. Dependence on Deflection, Mirror Size, and f-Number

In this section, the impact of parameters like pixel pitch or mirror deflection is investigated. Since most parameters are interdependent, a change in one parameter requires changes in the others in order to satisfy the system specifications. A summary of all input parameters relevant to the calculations is, therefore, given in Table 4.

**Figure 12:**
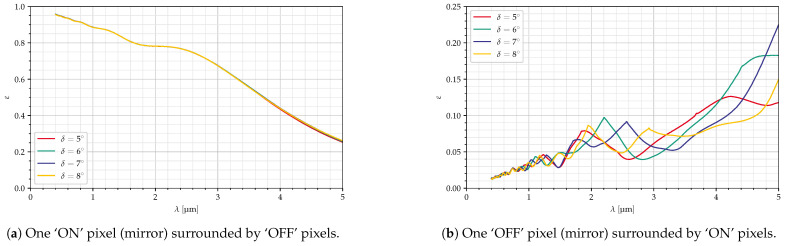
Dependency on deflection angle, p=30 μm, diagonal mirrors, all parameters in Table 4.

Figure 12 shows the efficiency for ‘ON’ and ‘OFF’ pixels for mirrors of 30 μm size and different deflection angles. Since the blaze wavelength depends on the deflection angle, one would expect higher efficiencies for ‘ON’ pixels and larger angles, especially at larger wavelengths. For ‘OFF’ pixels, ε should decrease with the deflection angle.

However, for ‘ON’ pixels, the curves are almost identical, indicating that the limiting factor is not the deflection (and thus the blaze wavelength) but the specifications for the optical setup, as discussed in Section 5.1. For ‘OFF’ pixels, the influence of the deflection angle can be seen more clearly; the curves are stretched out along the *x*-axis for larger angles (i.e., larger blaze wavelengths), but in general, they are still quite similar to each other.

**Figure 13:**
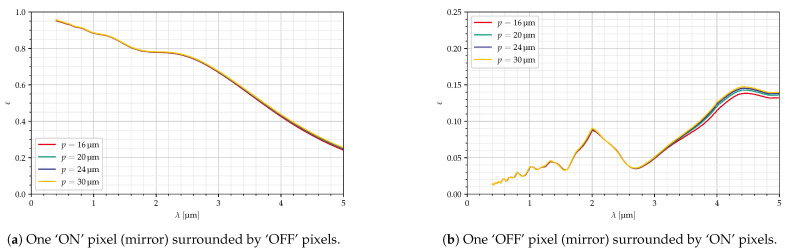
Dependency on mirror size, $N_{\mathrm{collect}}$, δ adjusted for *p*, diagonal mirrors, all parameters in Table 4.

For the curves in Figure 13, the mirror size was changed, while the deflection angle was adjusted to reach the same blaze wavelength ($\lambda_{B}=4\;\mu m$), and the collection optics focal length was changed according to Equation (2), resulting in different f-numbers. Here, all the curves are almost identical.

**Figure 14:**
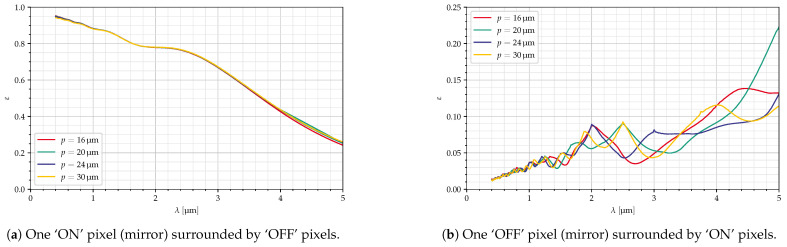
Dependency on the mirror size, δ=10.35°, $N_{\mathrm{collect}}$ adjusted for *p*, diagonal mirrors, all parameters in Table 4.

In Figure 14, the mirror size was changed while keeping the deflection angle constant (δ=10.35°, which leads to $\lambda_{B}=4\;\mu m$ at p=16 μm). The f-number was again adjusted for the mirror size. This means that larger mirrors have a larger blaze wavelength, and the result is similar to Figure 12.

**Figure 15:**
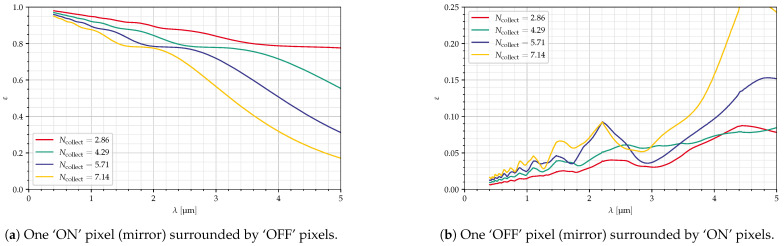
Dependency on $N_{\mathrm{collect}}$, δ=6°, p=30 μm, diagonal mirrors, all parameters in Table 4.

For Figure 15, the f-number was changed, while the mirror size and deflection were kept constant. It can be seen that, for smaller f-numbers, the ‘ON’ efficiency is much larger and less dependent on the wavelength, while the ‘OFF’ efficiency is much smaller. However, the configurations with the smaller f-number in this chart result in larger values for the GSD, see Table 4.

**Figure 16:**
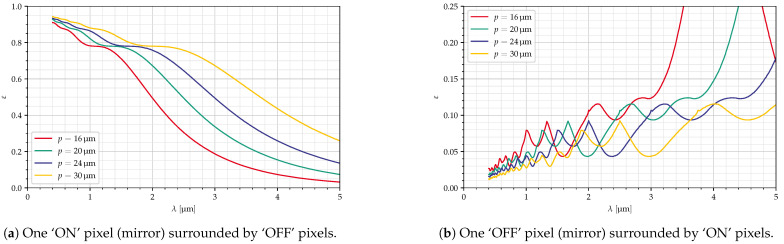
Dependency on mirror size, $f_{\mathrm{collect}}=6.17$, δ=6°, diagonal mirrors, all parameters in Table 4.

Figure 16 shows the dependency on mirror size, while the deflection angle and the f-number were kept constant. Here, we see a stretching out of the curves (both ‘ON’ and ‘OFF’ pixels) along the *x*-axis. The smaller pixels cannot be used at larger wavelengths, but, as Table 4 shows, they yield a smaller GSD.

To sum up this section, it can be said that the efficiencies are mainly determined by the system specifications, while the mirror architecture has relatively little influence as long as the conditions defined in Section 5.1 are met.

Also, there seems to be a trade-off between wavelength range and GSD so that, with the current system specifications, it will be difficult to have both a larger wavelength range and a small GSD. Some options for changes will be explored in Section 5.7.

### 5.6. Mirror Binning vs. Larger Mirrors

In this section, we investigate whether, for a given optical system and mirror array, mirror binning, i.e., the combination of several mirrors into one logical pixel or micropixel is feasible.

In this case, interference effects within a pixel become more complex. In order to simulate this, not a single Airy disk in the center of the pixel was considered but, rather, an array of Airy disks (10×10) distributed evenly over the micropixel. The Fourier transform, see Equation (22), was performed for each Airy disk, and the resulting intensities were added.

**Figure 17:**
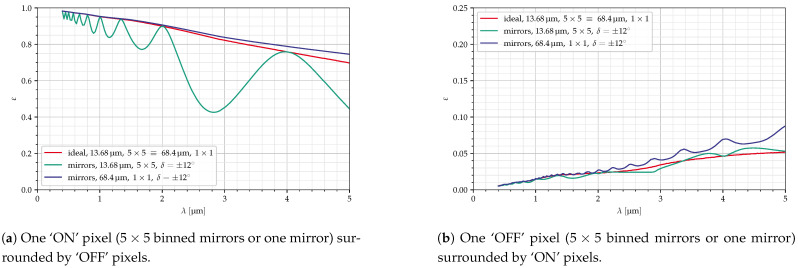
Diffraction efficiency for different micropixel sizes, $N_{\mathrm{collect}}=2.35$.

Figure 17 shows the efficiencies of an ideal modulator (which absorbs all the light on the ‘OFF’ pixels instead of reflecting it), a micropixel of 5×5 small mirrors, a micropixel of one large mirror with a reduced deflection angle (to achieve the same blaze wavelength), and a micropixel of one large mirror with the same deflection angle as the small mirrors.

These combinations cannot, in reality, be used since the resulting GSD is much too large (GSD≈3000 for $N_{\mathrm{collect}}=2.35$). These calculations are shown here because the curve for the binned micropixel shows an additional effect. The ‘ON’ efficiency shows a periodic change (with maxima at $n/\lambda_{B}$), resulting in very low values at some wavelengths.

### 5.7. Smaller GSD/Larger Wavelength

It has been stated above that, with the current specifications, it will be difficult to reach large wavelengths, as well as a small GSD. Here, we will explore what would have to be changed to achieve a better performance.

**Figure 18:**
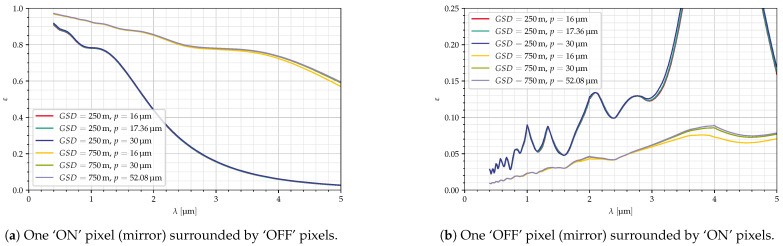
Efficiencies for modified system specifications (GSD) as in Table 5.

The first half of Table 5 shows six sets of parameters to either improve the GSD or increase the maximum wavelength that can be used. Figure 18 shows the resulting efficiencies.

**Table 5:** Parameters for calculation of trade-off between GSD and operational spectral range, Figure 18 (violated specifications in red).

Parameter	Unit	Smaller GSD			Larger $\lambda_{\mathrm{high}}$		
type		diag	diag	diag	diag	diag	diag
*p*	[μm]	16	17.36	30	16	30	52.08
$\lambda_{B}$	[μm]	4	4	4	4	4	4
δ	[°]	10.35	9.51	5.43	10.35	5.43	3.12
*d*	[μm]	2.03	2.03	2.01	2.03	2.01	2
$D_{\mathrm{Airy}}$	[μm]	64.2	69.7	120.5	21.4	40.2	69.7
$D_{\mathrm{Airy}}/p$		4.02	4.02	4.02	1.34	1.34	1.34
$N_{\mathrm{collect}}$		6.58	7.14	12.34	2.19	4.11	7.14
$f_{\mathrm{collect}}$	[m]	2.3	2.5	4.32	0.77	1.44	2.5
$d_{\mathrm{collect}}$	[m]	0.35	0.35	0.35	0.35	0.35	0.35
GSD	[m]	250	250	250	750	750	750
		$\lambda_{\mathrm{high}}<2\;\mu m$			$\lambda_{\mathrm{high}}>4\;\mu m$?		
$\varepsilon_{w}/\varepsilon_{b}\left(\lambda =4\;\mu m\right)$		0.11	0.11	0.11	9.93	8.60	8.27

If we want a GSD of 250 m instead of 500 m without changing the maximum focal length of the collection optics, the maximum mirror size is reduced to 17.36 μm. Then, all specifications are met with the exception of the maximum wavelength.

With respect to the efficiency, in the considered conditions, the mirror size is of very little relevance.

## 6. Summary and Conclusions

In this paper, we have shown that it is possible to design a micromirror array for use in compressive sensing that will work within the preliminary system specifications given in this paper. Based on these simulations, we would like to propose a scalable concept for a space-oriented SLM.

The mirrors can have a size in the range of about 10 μm to about 30 μm. Smaller mirrors offer an advantage in that they require smaller optics. On the other hand, the deflection angles for smaller mirrors would have to be larger while, at the same time, it would be more difficult to reach these large angles for smaller mirrors since the address electrodes and, therefore, the electrostatic forces would be smaller. Also, we would like to develop a device that can work in the MWIR and for other space applications, as well as the one discussed here and addressed in the SURPRISE project.

Therefore, we would prefer mirrors of 16 μm or larger, although we will continue to investigate the feasibility of manufacturing large-deflection mirrors of different sizes.

Although the simulations here show a slight advantage for the orthogonal mirrors (Figure 10), these results only apply to the optical setup in the SURPRISE concept, and since the aim is to design a device that can be used for other applications in space as well, we still favor the diagonal mirrors because of their low intensities for deflections larger than λ/2 (Figure 6). Furthermore, for diagonal mirrors, the distances between the 0th and 1st orders are larger than for orthogonal mirrors (Figure 9), which is an advantage in the practical implementation of the system.

Table 6 shows a summary of the parameters for a group of devices that meet the requirements of the SURPRISE project but also have potential for use in other space-related applications, like, for example, multi-object spectroscopy (MOS) [25] or grayscale optical correlators [26].

## Figures and Tables

**Table 1:** Comparison of micromirror actuation modes.

Force Equilibrium Actuation Mode	Mechanical Stop Mode
non-contact	contact
can be adjusted to different tilt angles	fixed tilt angle
no surface contact, thus more suitable for space according to the literature [13]	contacting surfaces (may lead to sticking phenomena)
more flexibility for other space applications, first successful environmental tests with IPMS SLM available [16]	first successful space qualifications tests with DMD available [17]
analog and binary deflection modes addressable	only binary deflection mode addressable

**Table 2:** Preliminary basic system specifications used for the calculations in this paper.

Name	Symbol	Minimum	Maximum
Ground sampling distance	GSD		500 m
Orbit height	$h_{\mathrm{orbit}}$	36.000 km	
Collection optics focal length	$f_{\mathrm{collect}}$		2.5 m
Collection optics diameter	$d_{\mathrm{collect}}$		0.35 m
Collection optics f-number	$N_{\mathrm{collect}}$	2	
Pixel size	*p*	9.72 μm (at *d*_collect,max_)	34.7 μm
Lower limit of operational spectral range	$\lambda_{\mathrm{low}}$		0.4 μm
Higher limit of operational spectral range (higher values would be desirable, but since they would be very difficult to achieve with current mirror technologies, the current value was set to a preliminary minimum)	$\lambda_{\mathrm{high}}$	4 μm	

**Table 3:** Deflection for minimum and maximum pixel pitches at blaze wavelength $\lambda_{B}=4\;\mu m$.

Pixel Size	Diagonal	Orthogonal
$p=p_{\min}=9.72\;\mu m$	δ=17.79°	δ=12.15°
	d=2.10 μm	d=1.02 μm
$p=p_{\max}=34.72\;\mu m$	δ=4.69°	δ=3.31°
	d=2.01 μm	d=1.00 μm

**Table 6:** Space-oriented SLM options.

Spectral range	From VIS to MWIR (up to 4 or 5 μm)
SLM actuation mode	Binary or analog
Tilt angle adjustment	Force equilibrium, i.e., non-contact mode
Mirror type	Diagonal
Mirror size, tilt angle	From [16 μm, ≥10°] to [30 μm, ≥5°]
Array size	From a few thousand up to several millions of micromirrors
